# DNA methylation changes in Down syndrome derived neural iPSCs uncover co-dysregulation of ZNF and HOX3 families of transcription factors

**DOI:** 10.1186/s13148-019-0803-1

**Published:** 2020-01-08

**Authors:** Loora Laan, Joakim Klar, Maria Sobol, Jan Hoeber, Mansoureh Shahsavani, Malin Kele, Ambrin Fatima, Muhammad Zakaria, Göran Annerén, Anna Falk, Jens Schuster, Niklas Dahl

**Affiliations:** 1 Department of Immunology, Genetics and Pathology, Science for Life Laboratory, Uppsala University, Box 815, SE-751 08 Uppsala, Sweden; 2 Department of Neuroscience, Karolinska Institutet, Stockholm, Sweden

**Keywords:** Down syndrome, Induced pluripotent stem cells, DNA-methylation, Neurogenesis, Transcription factors, Gene expression

## Abstract

**Background:**

Down syndrome (DS) is characterized by neurodevelopmental abnormalities caused by partial or complete trisomy of human chromosome 21 (T21). Analysis of Down syndrome brain specimens has shown global epigenetic and transcriptional changes but their interplay during early neurogenesis remains largely unknown. We differentiated induced pluripotent stem cells (iPSCs) established from two DS patients with complete T21 and matched euploid donors into two distinct neural stages corresponding to early- and mid-gestational ages.

**Results:**

Using the Illumina Infinium 450K array, we assessed the DNA methylation pattern of known CpG regions and promoters across the genome in trisomic neural iPSC derivatives, and we identified a total of 500 stably and differentially methylated CpGs that were annotated to CpG islands of 151 genes. The genes were enriched within the DNA binding category, uncovering 37 factors of importance for transcriptional regulation and chromatin structure. In particular, we observed regional epigenetic changes of the transcription factor genes *ZNF69*, *ZNF700* and *ZNF763* as well as the *HOXA3*, *HOXB3* and *HOXD3* genes. A similar clustering of differential methylation was found in the CpG islands of the *HIST1* genes suggesting effects on chromatin remodeling.

**Conclusions:**

The study shows that early established differential methylation in neural iPSC derivatives with T21 are associated with a set of genes relevant for DS brain development, providing a novel framework for further studies on epigenetic changes and transcriptional dysregulation during T21 neurogenesis.

## Background

Trisomy for chromosome 21 (T21) causes Down syndrome (DS) and is a leading cause of intellectual disability with an incidence of approximately 1 in 750 live births [1]. The supernumerary chromosome 21 causes major regional brain abnormalities and a broad range of distinct clinical features [2–4]. We and others have previously shown global gene expression changes in neural cells and brain specimens with T21 [5–10] and it is now generally accepted that DS is the result of complex transcriptomic changes induced by a genomic imbalance of human chromosome 21 (HSA21) [5–10]. While these earlier reports have shown several dysregulated genes in neural cells and brain tissues with T21, the precise mechanisms mediating the genome-wide transcriptomic alterations are still largely unknown.

Methylation of DNA constitutes a potent epigenetic regulatory mechanism for gene expression of fundamental importance for normal embryonic development and for postnatal health [11]. Methylation of promoter regions is associated with condensed inactive chromatin and gene silencing whereas methylation within the body of genes may be correlated with splice site usage and increased gene expression [12, 13]. Because the anatomical and cellular brain abnormalities in DS are established at birth, the identification of mechanisms and pathways along the differentiation of trisomic neural cells are important. Prior studies of DNA-methylation patterns in pre- and postnatal brain specimens with T21, as well as in orthologous mice models, have shown alterations across the entire genome when compared to matched euploid tissues [14–16]. Slight generalized hypermethylation in DS fetal cortex has been reported [14, 15] accompanied by overexpression of the DNA methyltransferase 3L, encoded on chromosome 21 [15]. A more recent study, performed by whole-genome bisulfite-sequencing on adult DS brain specimens, reported no differences in global methylation profiles when compared to controls. However, the same study showed enrichment for differentially CpG islands in DS brain samples [17]. While these independent studies have brought important knowledge on epigenetic changes in developing and adult DS brains, the correlation to the global transcriptional changes remains elusive.

The limited access to brain specimens has made neural derivatives from induced pluripotent stem cells (iPSCs) an attractive in vitro model of disease [18]. To date, one study has undertaken a DNA methylation analysis of iPSC with T21 [19] showing methylation differences when comparing trisomic and euploid iPSCs. However, the study was performed on undifferentiated iPSCs and thus not translatable to the developing brain. Noteworthy, the study confirmed enrichment for a subset of differentially methylated *HOX* genes in trisomic cells, a finding previously observed in the placenta, leukocytes, and buccal cells with T21 [20–23]. Herein, we set out to analyze the methylation pattern of all known CpG regions and promoters in trisomic and matched euploid iPSCs differentiated into the neural lineage. The iPSC-derived neural model used in this study has shown a transcriptional profile comparable to that of fetal brains at the early and mid-gestational stages, respectively [10]. We present herein the identification of CpGs regions and promoters across the genome with a consistent pattern of differential methylation pattern in T21 neural cells at two distinct stages of differentiation when compared to euploid cells. Further analysis of differentially methylated CpGs assigned to CpG islands (CGIs) uncovered enrichment of genes for DNA binding and transcriptional regulation. Our study shows the utility of iPSCs derivatives to bring insights into epigenetic mechanisms associated with transcriptional changes during T21 neurogenesis and the combined data provide a framework for further functional studies to interfere with DS brain development.

## Results

### Neural iPSCs derivatives with T21 show differentially methylated CpGs with uneven chromosomal distribution and hypomethylation of chromosome 21

**Fig. 1:**
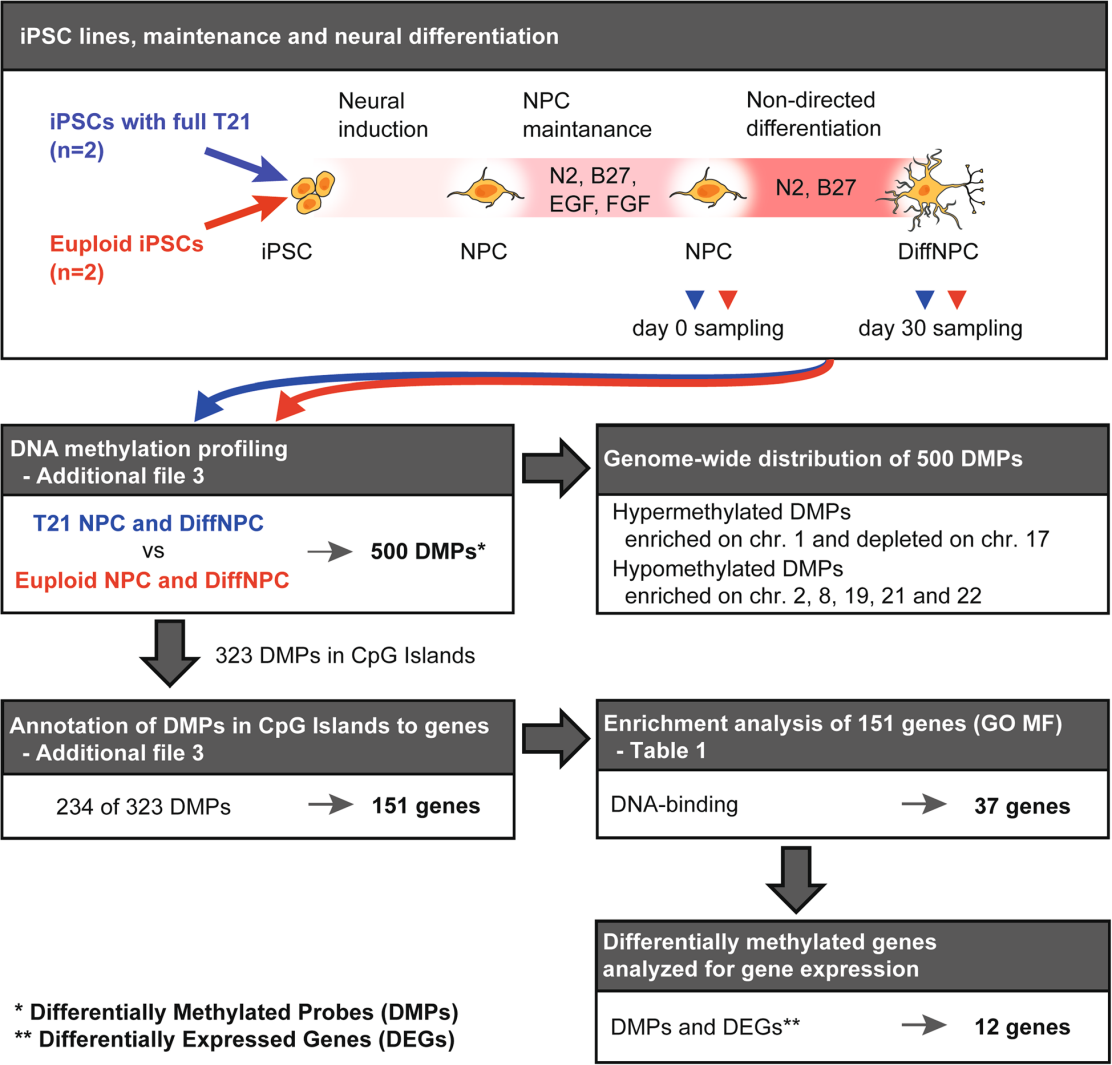
Overview of the study. Neural iPSC derivatives from two Down syndrome subjects with full trisomy 21 (T21) and two healthy (euploid) subjects were harvested at two stages of differentiation for DNA-methylation analysis of CpGs queried by probes on the 450K array (Illumina). Differentially methylated probes (DMPs) associated with T21 neural lines, and at two stages of differentiation, were assigned to CpG islands (CGIs) and genes. Subsequent enrichment analysis identified 37 genes that were subject to expression analysis

Genomic DNA for methylation analysis of known CpG regions and promoters across the genome was isolated from previously established neural iPSC cultures derived from one male and one female (DS1 and DS2) with characteristic DS features and a full T21, as well as from two age and gender-matched euploid donors (Ctrl1 and Ctrl2) [10]. The DNA was obtained from iPSC derived neural progenitor cells (NPCs) [24], and further differentiated for 30 days (DiffNPC) using an undirected protocol ([10]; Fig. 1). Staining and RNA sequence analysis of neural markers confirmed that the composition of major neural cell types was comparable in trisomic and euploid cultures, and at both differentiation stages (Additional file 1a, b). The transcriptional profiles at the NPC and DiffNPC stages correspond to that of different brain regions, including the hind- and midbrain, at early- and mid-gestation, respectively [10].

**Fig. 2:**
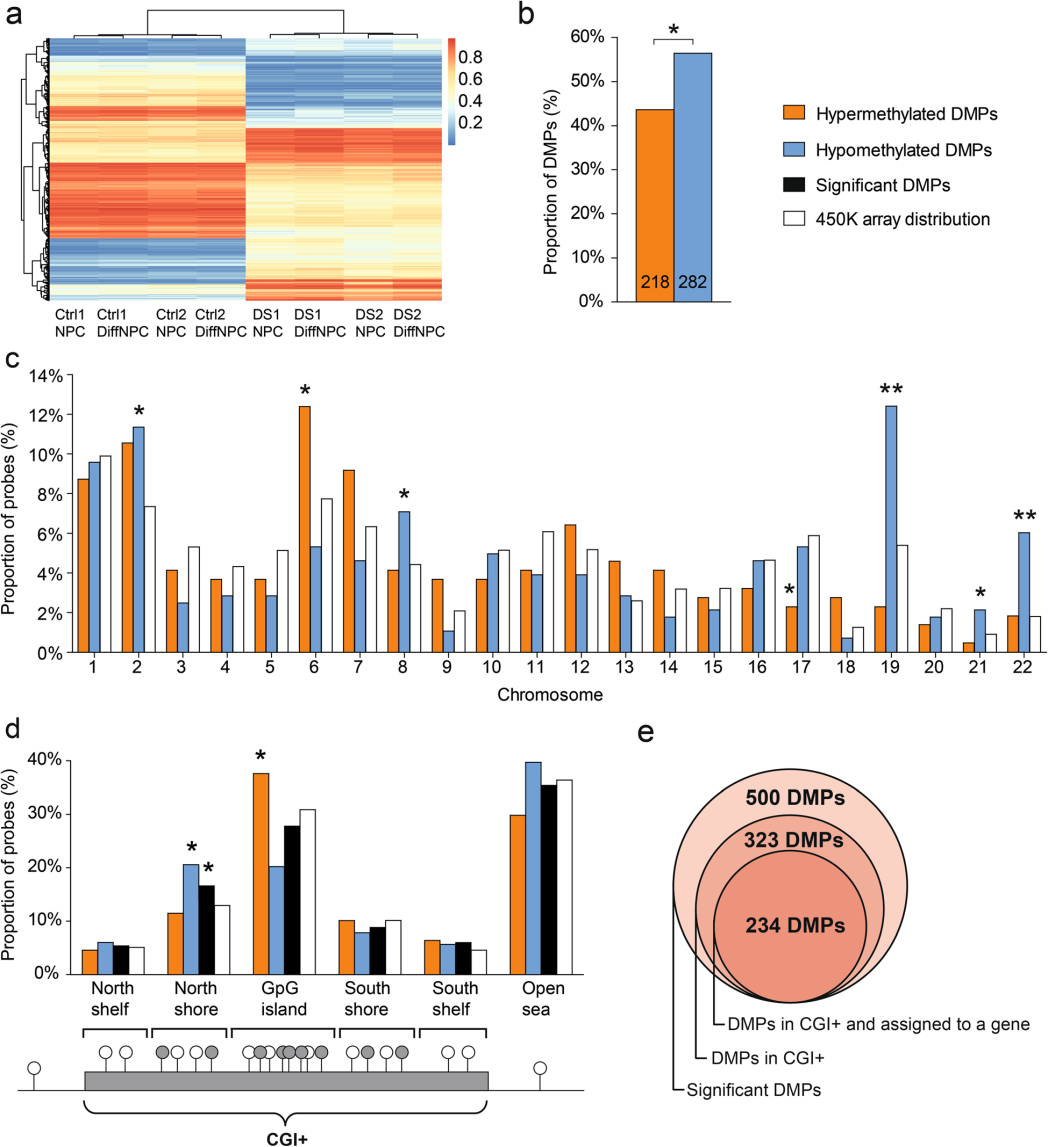
DNA methylation changes in iPSC neural derivatives with T21. **a** Heat map of 500 differentially methylated CpG sites in T21 and euploid neural cell lines. Hierarchical clustering of the 500 CpGs in euploid (Ctrl) and trisomic (DS) neural lines at the NPC and DiffNPC differentiation stages, respectively (differences > 15% of average beta values in Ctrl vs. DS lines). Methylation values are color-coded according to legend. **b** Bar chart represents the distribution of hypermethylated (orange bars) and hypomethylated DMPs (blue bars) among all 500 DMPs in T21 lines. An enrichment of hypomethylation in T21 neural lines is examined (*p* = 0.049). **c** Chromosomal distribution of hyper-and hypomethylated DMPs in T21 neural lines. White bars represent percentages of CpG sites queried on a particular chromosome, orange bars percentages of hypermethylated DMPs and blue bars the percentages of hypomethylated DMPs. Chromosome 1 is significantly (*p < 0.05) enriched for hypermethylated DMPs. Chromosome 17 is depleted of hypermethylated DMPs and chromosomes 2, 8, 19, 21, and 22 are enriched for hypomethylated sites (**p < 0.005). **d** The positions of CpGs called by DMPs are illustrated relative to CpG islands (CGIs) and flanking genomic regions compared to the average of 450K array. Bars represent distribution of hypermethylated DMPs (orange; *n* = *2*18), hypomethylated DMPs (blue; *n* = 282), all DMPs (black; *n* = 500) and 450K array coverage (white) in different genomic regions. Individual CpGs (grey and white circles) were classified based on their location relative to a CpG island (bottom). The proportion of differentially methylated probes in north shores are overrepresented (*p* = 0.016) and preferentially hypomethylated (*p* = 0.0003) when compared to the genome average. In contrast, CpG islands are enriched in hypermethylated CpGs (*p* = 0.034). **e** Euler diagram illustrates 500 DMPs in T21 neural iPSCs comprising 323 DMPs located within or close to a CpG island (CGI+) of which 234 DMPs are assigned to a gene

Previous studies of the fetal and adult cortex and cerebellum have indicated that at least 90% of CpGs are stably established at an early fetal stage [25, 26]. We therefore asked what CpGs exhibited a consistent differential methylation pattern in trisomic NPCs and trisomic DiffNPCs (*n* = 4) when compared to euploid NPCs and DiffNPCs (*n* = 4). To this end, we analyzed the DNA methylation signatures of all known CpG regions and promoters using the Illumina Infinium Human Methylation 450K Bead chip that interrogates > 480,000 CpG sites throughout the genome [27]. The probe call rate for the eight samples analyzed in our study varied between 99.64% and 99.97% (detection *p* value < 0.01). We retrieved *β* values (ratio of intensities between methylated and unmethylated alleles, range between 0 and 1) for 485,512 probes out of a total of 485,553 loci analyzed in all samples [27]. From the raw data of each sample, we prepared density plots of the *β* values for visual inspection (Additional file 2a). Quality assessment of the refined number of probes revealed a bimodal distribution of acquired data (Additional file 2b) consistent with previous reports [15, 28]. We then compared the assessed methylation data of trisomic NPCs and DiffNPCs (*n* = 4) with those of euploid NPCs and DiffNPCs (*n* = 4), respectively. Using this approach, we identified 500 differentially methylated probes (DMPs), corresponding to approximately 0.1% of all CpGs queried by the array, that clustered together in the four trisomic samples at both stages of neural differentiation (Additional file 3). This suggests only small differences in global methylation between the trisomic and euploid neural iPSC derivatives. Hierarchical clustering analysis further illustrated that the differential methylation pattern detected by the 500 probes was established during early neural differentiation (in NPCs) and remained into the DiffNPC stage (Fig. 2a). Among the 500 DMPs in trisomic neural cells, 218 (43.6%) were hypermethylated whereas 282 (56.4%) were hypomethylated (*p* = 0.049) (Fig. 2b). To further investigate the chromosomal distribution of the CpGs corresponding to the 500 DMPs, we determined the proportion of sites that were hyper- and hypomethylated, respectively, on each chromosome. Notably, several chromosomes in trisomic lines deviated from the expected distribution. The proportion of hypermethylated DMPs were enriched on chromosome 6 but depleted on chromosome 17 (*p* < 0.05) (Fig. 2c). On the other hand, chromosomes 2, 8, 19, 21 and 22 were enriched for hypomethylated sites (*p* < 0.05) (Fig. 2d). The enrichment of hypomethylated sites on chromosome 21 is consistent with previous findings in specimens from the fetal DS cortex [14, 15]. These observations suggest that the 500 DMPs, with a stable differential methylation pattern in trisomic NPCs and DiffNPCs, are unevenly distributed on chromosomes.

CpG islands (i.e., CGIs) and their flanking regions usually co-localize with gene promoters and are therefore more likely associated with transcriptional regulation [29–32]. We therefore investigated the methylation pattern of probes annotated close to a CGI, i.e., either within a CGIs [33] or at shores and shelves of a CGI (within 2 kb or 4 kb of a CGI, respectively), henceforth defined as a CGI+ (Fig. 2d) [34]. Regions outside a CGI+ are identified as open sea. Out of the 500 DMPs, 323 (65%) were annotated at a CGI+ (Fig. 2d, e). Specifically, 139 (28%) were located within a CGI; 83 (17%) in N-shores and 44 (9%) in S-shores; 27 (5%) in N-shelves and 30 (6%) in S-shelves. Out of the 500 DMPs, 177 (35%) were located in the open sea. The distribution of all 500 DMPs revealed enrichment in N-shores (*p* = 0.016) but was otherwise as expected (Fig. 2d). Furthermore, the DMPs in N-shores were found to be predominantly hypomethylated (*p* = 0.0003). In contrast, DMPs in the CpG islands (CGI) were mainly hypermethylated (*p* = 0.034) and with an expected distribution in the total number of DMPs. These data, indicating preferential hypermethylation of CGIs, are consistent with that previously reported in the developing human fetal brain [25]. To corroborate our methylation data from the 450K array, we used the bisulfite sequenced-based EpiTYPER assay for region-specific DNA methylation analysis of five CGIs+ called by DMPs [35]. The analysis of the selected CGI+ regions using the EpiTYPER assay confirmed differential methylation in T21 neural lines in all five regions (Additional file 4a–c).

Taken together, we identified 500 DMPs in DNA of trisomic NPCs and DiffNPCs when compared to the corresponding euploid cells. Out of these 500 DMPs, 323 were annotated to within or close to CpG islands (CGI+) (Fig. 2e, Additional file 5), suggesting a direct regulatory effect.

### Differentially methylated genes in neural iPSC derivatives are enriched in the DNA binding category of importance for transcriptional regulation

**Fig. 3:**
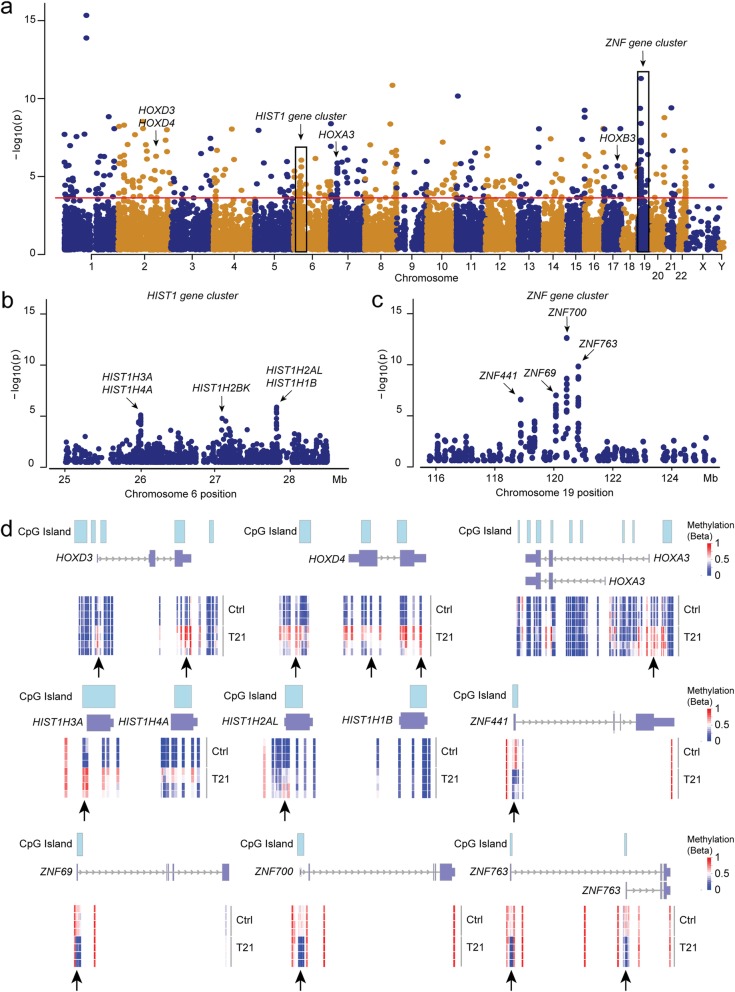
Chromosomal and regional distribution of differential methylation consistent in NPCs and DiffNPCs with T21. **a** Genomic coordinate dot plot (Manhattan plot) of CpGs detected by 500 DMPs in T21 neural NPCs and DiffNPCs showing methylation changes of known CpG regions and promoters and clustering at annotated genes (arrows and boxes). *X* axis represents chromosomes ranked by number, *Y* axis represents –log10 (*p* values) and the red line indicates significance level (Bonferroni; *p* < 5 × 10^−7^). **b**, **c** Zoom-in of boxed regions in **a** showing the hypo-methylated *HIST1* gene cluster on chromosome 6 (**b**) and the hyper-methylated *ZNF* cluster on chromosome 19 (**c**). **d** Details on the DMP pattern (arrows) of three *HOX* genes (top). Differential methylation of *HOXD3* and *HOXA3* genes are located in shores and shelves of CGIs. In contrast, differential methylation of genes belonging to the *HIST1*- and *ZNF*-clusters (mid and bottom) were specific to CGIs. Each DMP is colored according to methylation state (*β* values) ranging from low (blue) to high (red) methylation in T21 cells

CGIs are predominantly but not always associated with promoter regions or other regulatory regions of genes [32]. We therefore investigated if the 323 DMPs in CGI+ regions were located close to an annotated gene (i.e., within 1.5 kb or 200 bp from a transcriptional start site (TSS1500 or TSS200, respectively), in the 5′UTR, first exon, gene body or 3′UTR, of a gene) [36]. This approach uncovered 234 DMPs belonging to a differentially methylated CGI+ region of 151 annotated genes (Fig. 2e). These 151 genes were further analyzed for enrichment in functional categories using the Gene Ontology (GO) knowledge database. Using this approach, we identified a single functional category, namely DNA binding (GO:0003677; Enrichment 2.23; FDR 8.22E-03), that was significantly enriched for 37 genes (Table 1). Notably, the DNA binding category comprised multiple members of the *HOX*, *HIST1*, and *ZNF* family of genes encoding proteins important for transcription and chromatin structure (Fig. 3a). Specifically, our analysis identified hypermethylation of *HOXD4* and *HOX* group 3 family members (*HOXA3*, *HOXB3*, and *HOXD3*), distributed on chromosomes 2, 7, and 17 (Fig. 3a). The *HOX 3* genes are critical for cell fate determination and morphogenesis of several organs such as the brain [37–40]. We also identified hypomethylation of the five histone protein coding genes *HIST1H3A*, *HIST1H4A*, *HIST1H2BK, HIST1H2AL*, and *HIST1H3A* clustered on chromosome 6 (Fig. 3a, b). The *HIST1* genes encode proteins that are critical for chromatin structure and remodeling [41, 42]. Furthermore, out of 13 *ZNF* genes in the DNA binding category, four were hypomethylated (*ZNF69*, *ZNF441*, *ZNF700*, and *ZNF763*) and clustered within a 1 Mb region on chromosome 19 (Fig. 3a–c). The downstream targets of these transcription factors (TFs) are yet unknown, however, protein expression data from Human Protein Atlas (HPA version 18.1; proteinatlas.org) [43] show that three of these *ZNF* genes (*ZNF69*, *ZNF700*, and *ZNF763*) encode for proteins that are highly expressed in glial cells of adult cerebral cortex. Moreover, the differential methylation of the *HOX*, *HIST1*, and *ZNF* genes is confined to their CGI+ regions with a normal methylation pattern outside the CGI+ regions, further suggesting a regulatory impact (Fig. 3d). Additionally, the DNA-binding category was enriched for six genes (*SP5*, *SP3*, *NEUROD6*, *PAX5*, *CREBZF*, and *SIM2*) encoding TFs of importance for early neural differentiation, as well as a group of eight genes encoding DNA-binding proteins relevant for transcription (Table 1).

**Table 1:** List of 37 enriched genes belonging to the DNA binding category (GO:0003677)

Group	Gene name	CGI position (hg19)	State	# DMP	DEG
**HOX proteins**	*HOXD4*	chr2:177014948-177015214	Hyper	2	
	*HOXD3*	chr2:177029413-177029941	Hyper	1	Up
	*MEIS1*	chr2:66672431-66673636	Hypo	3	
	*HOXA3*	chr7:27163819-27164098	Hyper	3	Up
	*HOXB3*	chr17:46631800-46632212	Hyper	1	Up
**HIST1 proteins**	*HIST1H3A*	chr6:26020671-26021125	Hyper	1	
	*HIST1H4A*	chr6:26020671-26021125	Hyper	3	
	*HIST1H2BK*	chr6:27107138-27107394	Hyper	1	
	*HIST1H2AL*	chr6:27833120-27833406	Hyper	4	
	*HIST1H1B*	chr6:27835190-27835461	Hyper	1	
**ZNF transcription factors**	*PRDM16*	chr1:3102540-3103352	Hypo	2	
	*ZNF512*	chr2:27805754-27806078	Hyper	1	Down
	*ZNF518B*	chr4:10458129-10459353	Hyper	1	Down
	*CXXC5*	chr5:139040819-139041028	Hypo	1	Up
	*FEZF1*	chr7:121943867-121944538	Hypo	3	
	*TRPS1*	chr8:116660432-116660747	Hyper	1	
	*ZNF263*	chr16:3332472-3333847	Hypo	1	
	*ZNF397OS*	chr18:32847284-32848130	Hyper	1	
	*ZNF441*	chr19:11877720-11878280	Hypo	1	Up
	*ZNF69*	chr19:11998804-11999131	Hypo	10	Up
	*ZNF700*	chr19:12035899-12036433	Hypo	9	Up
	*ZNF763*	chr19:12076029-12076366	Hypo	9	
	*ZNF529*	chr19:37095680-37096589	Hyper	1	
**Transcription factors**	*SP5*	chr2:171569877-171573904	Hypo	2	
	*SP3*	chr2:174828330-174830617	Hypo	1	
	*NEUROD6*	chr7:31375845-31376542	Hyper	1	
	*PAX5*	chr9:36985986-36986924	Hypo	1	Up
	*CREBZF*	chr11:85374872-85376234	Hypo	1	
	*SIM2*	chr21:38079941-38081833	Hypo	1	
**DNA-binding proteins**	*COLEC11*	chr2:3683029-3683290	Hypo	1	
	*WRNIP1*	chr6:2765203-2766775	Hyper	2	
	*JRK*	chr8:143750759-143751448	Hypo	2	
	*FBXO21*	chr12:117627650-117628488	Hypo	2	Up
	*KDM2B*	chr12:121975028-121976140	Hyper	1	
	*POLE*	chr12:133249979-133250243	Hypo	1	Down
	*WNT1*	chr12:49371690-49375550	Hyper	2	
	*SMC1B*	chr22:45809191-45809953	Hypo	2	

Genes annotated to a CGI+ with at least 1 DMP. Columns denote groups of encoded proteins, position of CGIs, methylation state, number of DMPs and differential expression of genes (DEG; *Up* upregulated, Down downregulated; *p* < 0.05)

As methylation of CGIs and flanking regions are likely to cause transcriptional repression of a nearby gene, we asked if the altered methylation pattern of CGI+ of the 37 enriched genes correlated with differential expression. To this end, we revisited our gene expression data from T21 and euploid lines obtained from the DiffNPC differentiation stage [10]. The analysis revealed that 12 out of the 37 genes (32%) were differentially expressed (*p* < 0.05) in T21 DiffNPCs (Table 1). To validate the gene expression data retrieved from RNA sequencing, we selected three differentially expressed genes (DEGs; *ZNF700*, *HOXA3*, *HOXB3*) for RT-qPCR analysis that confirmed an altered gene expression (Additional file 6). Notably, three *HOX3* genes showed increased expression despite hypermethylated CGI+ regions (Table 1). However, the DMPs in these CGI+ regions were predominantly annotated in shores and not the CGIs themselves.

Taken together, these data show enrichment of differential methylation in CGI+ that are linked to genes encoding for proteins regulating transcription and chromatin remodeling relevant for DS brain development. Given the genome-wide transcriptional perturbations in the DS brain, our data suggest specific epigenetic changes that may contribute to the altered gene expression profile during early neural development in T21 cells.

## Discussion

We present herein, and to our knowledge, the first DNA methylation analysis of neural iPSCs derivatives with T21. When compared to matched euploid cells and previous reports on methylation data obtained from DS brain specimens, the results support our trisomic neural model to be relevant for epigenetic changes in early DS neurogenesis. Despite the low number of biological replicates in our study we identified 500 DMPs, using genomic DNA of neural cells with T21, showing a consistent pattern at two distinct time points of neural differentiation. Importantly, the DMPs were annotated in, or close to, CpG islands of 151 genes enriched in factors important for DNA binding, transcriptional regulation, and chromatin structure.

The 500 DMPs correspond to a small fraction (0.1%) of all CpGs analyzed. This is in line with a small proportion of DMPs identified in DS cortical samples from previous studies [14, 44]. However, the tendency towards hypomethylation (56%) of CpGs at the two stages of neural differentiation in our study differs slightly from the tendency towards global hypermethylation observed in fetal and adult DS cortex [14, 15, 17]. Cell type-specific methylation patterns develop already at the neural progenitor stage [45] and cell-specific differences have been identified between glia and neurons that are characteristic for DS cortex [44]. A possible reason for the global increase in hypomethylated DMPs in our iPSC-derived model of DS neurogenesis may thus be due to composition of cell types in the neural cultures. The undirected differentiation protocol leads to a considerable proportion of cells representing mid- and hindbrain precursors [10] that are less abundant in the developing cortex. However, the chromosomal distribution of DMPs in our trisomic neural cells showed a significant enrichment of hypomethylation on chromosome 21 consistent with other studies [14, 15]. We also identified hypomethylation of chromosomes 2, 8, 19, and 22 whereas chromosomes 6 were enriched and 17 depleted with hypermethylated probes. Uneven chromosomal distribution of DMPs has been observed across a variety of cells and tissues with T21 [14, 15, 46, 47]. The reason for this is unclear and it may be hypothesized that the genomic imbalance in T21 affects the chromosomal organization in 3D, with distorted interactions between certain genomic regions, resulting in a skewing of the chromosomal and subchromosomal DNA methylation pattern.

Among the DMPs, we identified 234 probes called CpGs adjacent to 151 genes and thus with a more likely effect on transcription. This number is comparable to that previously reported for differentially methylated genes in glia and neurons from fetal and adult DS cortical samples [44]. Furthermore, the differentially methylated CGIs in our trisomic neural cells were preferentially hypermethylated consistent with previous studies of the frontal cortex and glia cells in DS brain specimens [15, 17, 44]. Interestingly, we noted that several differentially methylated CGI+ regions were clustered close to discrete sets of genes distributed on various chromosomes. These gene sets were further highlighted in our enrichment analysis that uncovered altogether 37 differentially methylated genes within the DNA binding category (GO:0003677). In particular, we uncovered families of genes such as homeobox TFs genes represented by *HOXD4*, *HOXA3*, *HOXB3*, and *HOXD3* essential for the development of the hindbrain [37–40] and mesenchymal neural crest-derived structures [48]. Three of these genes showed increased expression in neural T21 cells that were associated with hypermethylation mainly in their shores and shelves (Additional file 3; Fig. 3d). Hypermethylation in shores and shelves around CGIs are sometimes, and in contrast to hypermethylation of CGIs, positively correlated to the expression of genes [49, 50]. Effects on pathways for hindbrain were further supported by differential methylation of the *MEIS1* gene encoding a homeobox related TF [51]. In mice, the paralogous *Hox3* genes are important for the delineation of the different rhombomeres in the developing hindbrain and for the formation of distinct neuronal lineages within each rhombomere [37]. These observations in our T21 neural lines are consistent with some of the anatomical brain abnormalities in DS such as cerebellar hypoplasia [4, 52]. Furthermore, differential methylation in *HOXA3* and/or *HOXD3* have previously been observed in glia cells from DS fetal brains [44], peripheral blood leukocytes and fibroblasts of DS patients, and undifferentiated iPSCs with T21 [19, 47] suggesting dysregulated *HOX* genes to play an important role for a plethora of features characteristic for DS from early stages of development.

Furthermore, we observed hypermethylation of CGIs in the *HIST1* gene cluster comprising *HIST1H4A*, *HIST1H3A*, *HIST1H2BK*, *HIST1H2AL*, and *HIST1H1B* on chromosome 6. The cluster constitutes 80% of genes encoding the canonical histone proteins (H2A, H2B, H3, and H4) and the linker histone (H1) [42] that are of fundamental importance for chromatin structure. Histone modifications regulate the accessibility of chromatin to various TFs and the activity states of DNA [53]. Interestingly, several neurodevelopmental disorders, for example, the ATRX syndrome [54] and Rett syndrome [55], are associated with mutations in genes important for chromatin structure and remodeling. Furthermore, dysregulation of several genes encoding Histone proteins was recently identified in a neurodevelopmental model of Dravet syndrome [56]. Given the role of Histone proteins for chromatin remodeling, differential methylation of the *HIST1* gene cluster may suggest a downstream effect on chromatin structure and transcriptional dysregulation in trisomic cells. We also identified differential methylation of 13 genes encoding Zinc-finger transcription factors. Four out of these genes belong to a single cluster on chromosome 19 (Fig. 3a, c) with hypomethylated CGIs. Analysis of RNA sequencing data and by RT-qPCR showed that three of these clustered *ZNF*-genes had significantly reduced expression levels (Fig. 3d, Table 1) [57]. These Zink-finger TFs are highly expressed in the central nervous system but their precise role during development is yet unknown. Zinc-finger proteins belong to the most abundant class of proteins in the human proteome but the majority remain uncharacterized as well as their downstream targets [58, 59]. However, the ability of zink-finger domains to interact with DNA and RNA suggests this family of proteins have a role in a broad range of functions beside transcriptional regulation. Furthermore, our enrichment analysis identified two additional groups of genes encoding six different transcription factors and eight DNA interacting proteins (Table 1). One of the genes is *SIM2* within the DS critical region on chromosome 21 [9] encoding a TF important for cell fate determination [60]. Interestingly, a prior study showed that *SIM2* expression in fetal brain co-localizes with regions associated with DS pathology [61]. Taken together, the differentially methylated CGI+ regions identified in our trisomic neural model highlights genes and gene families of importance for transcriptional regulation and chromatin structure, providing further mechanistic insights into DS neurogenesis.

The association of DMPs with genes that are enriched in the DNA-binding category and for transcriptional regulation in our T21 neural model is in line with the previously and well documented global transcriptional changes of the DS brain [8]. It has been proposed that epigenetic mechanisms are critical for the transcriptional perturbations in DS [9] and previous reports have shown enrichment for DMPs at certain TF binding motifs in DS brain specimens [17, 44]. Given the genome-wide gene expression changes in DS brains, the differentially methylated genes and gene families identified in our study suggest important novel links between the genomic unbalance caused by T21 and the global transcriptional dysregulation in DS brains. We recently showed that the transcriptional dysregulation in iPSC-derived neural cells with T21 is confined to major functional clusters [10]. While this previous study suggested major functional clusters to be perturbed in T21 neural cells, DNA-binding was not identified as a disrupted functional category. The present study thus adds to previous reports by showing marked differential methylation confined to a set of genes important for transcriptional regulation and chromatin remodeling. In support of these findings, one-third of the genes enriched in the DNA binding category were found differentially expressed.

The vast majority of CpGs in the human genome are predominantly methylated as 5-methylcytosine (5mC; methylation at the 5-carbon position) [11]. Hydroxylation of 5mC results in 5hmC, a demethylation intermediate shown to be present in 4% of mammalian CpGs [62, 63]. It has been suggested that 5hmC may be a regulator for the elimination of cytosine methylation during development [63, 64]. Unfortunately, the commonly used methods to study DNA methylation based on bisulfite treatment do not distinguish between 5mC and 5hmC [62] why different detection methods are required to resolve the potential regulatory role of 5hmC in neurogenesis.

Intellectual disability is a predominant feature in DS and likely related to several distinct cell populations. The human brain forms highly diverse neuronal subpopulations presumably characterized by distinct methylation and expression profiles. Indeed, it has been demonstrated that the methylation pattern is different when comparing major neuronal subtypes in both mice [45] and humans [44]. Furthermore, a previous study reported that methylation patterns clustered differently when comparing distinct cortical brain regions as well as when comparing specific brain regions from DS patients with those from healthy subjects [44]. In our neural cultures, the cell populations are mixed with a predominance of mid- and hindbrain neural cells [24]. Given the heterogeneity of neural cells in our culture system, the methylation changes associated with T21 may therefore reflect a sum of different methylation signatures related to different neural subpopulations. Furthermore, our study focused on DMPs showing a consistent pattern at two stages of neural differentiation corresponding to early and mid-gestation. This approach selected for DMPs that are stably established at an early differentiation stage. However, previous studies on fetal brain tissues have shown only minor changes in the overall differential methylation pattern with gestational ages [25, 44] supporting that the majority of DMPs detected in our iPSC derived neural cells with T21 reflects an early established and stable differential methylation pattern.

## Conclusions

Our study suggests that iPSCs with T21 differentiated into neural lineages may serve as a translatable model for the identification of epigenetic changes that are associated with transcriptional perturbations in DS neurogenesis. The differentially methylated regions identified in our trisomic neural model highlights specific genes and gene families of importance for transcriptional regulation and chromatin structure. Taken together, the data provides a framework for further studies on epigenetic variation and specific factors mediating transcriptional changes downstream of chromosome 21 during DS brain formation. While our model shows extensive promise for further understanding of molecular mechanisms behind perturbed and early neurogenesis in DS, the undirected protocol used to generate neural cells from iPSCs is not translatable to the cortex but rather to the entire fetal brain at early stages of development [10]. Further investigations of differential methylation in functionally distinct neuronal cell populations with T21, preferably connected to transcriptome profiles, are now required to clarify the role of DNA-methylation changes for perturbed neurogenesis and ultimately for DS brain formation. More complex models of human neural differentiation using, e.g., 3D organoids from iPSCs with T21, build up by a mixture of cell types that are analyzed individually, may add important information in this context.

## Methods

### iPSC lines, maintenance, and neural differentiation

We previously established iPSCs with HSA21 by transducing fibroblast cells from one male and one female (DS1 and DS2) with characteristic DS features and a full T21, as well as from two age and gender-matched healthy individuals (Ctrl1 and Ctrl2) [10]. Fibroblasts were reprogrammed using a non-integrating method with CytoTune™-iPS 2.0 Sendai Reprogramming Kit (ThermoFisher Scientific, cat no: A16517). Standard karyotype analysis confirmed T21 or euploidy in iPSC derived from DS patients and healthy donors, respectively. Selected iPSC lines were induced to neural progenitor cells (NPC) as described [10]. The NPCs were grown on 0.1 mg poly-L-ornithine (Sigma-Aldrich, cat no.: P4957-50ML) and 1 μg/mL laminin (Sigma-Aldrich, cat no: L2020-1MG)-coated plates in DMEM/F12 GlutaMAX medium (Gibco, cat no.: 31331028) supplemented with 10 ng/mL rhFGF-basic (R&D Systems, cat no.: 233-FB-010), 10 ng/mL rhEGF (R&D Systems, cat no.: 236-EG-200), B27 supplement (1:1000, Gibco, cat no.: 08-0085SA), N2 supplement (1:100, Gibco, cat no.: 17502-048), and 1% penicillin/streptomycin (Gibco, cat no.: 15140-122). Self-renewing NPCs derived from the four donors were differentiated for 30 days into DiffNPCs using an undirected protocol (Fig. 1) as described [10, 24].

### Immunofluorescence

Immunofluorescent staining of the trisomic and euploid neural cultures was performed using standard techniques [65] at both differentiation stages as described previously [10]. The primary antibodies against Nestin (1:100, R&D Systems, cat no.: MAB1259), Pax6 (1:100, BioLegend, cat no.: 901301), β-III-tubulin (1:80, Sigma-Aldrich, cat no.: T2200), GFAP (1:500, Sigma-Aldrich, cat no.: G3893-.2ML), and Vimentin (1:500, Abcam, cat no.: ab92547) were incubated overnight at 4 °C. Subsequently, the cells were stained with α-mouse IgG Alexa Fluor 488 (1:10000, Thermo Fisher Scientific, cat no.: A-11001) and α-rabbit IgG Alexa Fluor 555 (1:10000, Thermo Fisher Scientific, cat no.: A-21406) for 1.5 h at room temperature. Nuclei were stained with DAPI (1 μg/mL, Sigma-Aldrich, cat no.: D8417). Visualization was performed on a Zeiss 510 confocal microscope.

### Heat-map profiling of neural genes

A set of known markers for neuroepithelial cells, radial glia, oligodendrocytes, astrocytes, microglia, immature neurons, mature neurons, glutamatergic neurons, and GABAergic neurons were selected. Expression of markers was retrieved and analyzed from RNA sequencing data (log2(counts)) of trisomic and euploid derived neural cultures at the NPC and DiccNPC stages of differentiation [10]. The Pheatmap package in R was used to generate heatmap profiles of the expressed marker genes.

### DNA extraction and microarray analysis

Total Genomic DNA was isolated using NucleoSpin® Tissue kit (Macherey-Nagel, cat no.: 740952.250) following the manufacturer’s instructions. Extracted genomic DNA was treated with sodium bisulfite using EZ-96 DNA methylation Gold kit (Zymo Research, cat no.: D5007). Assessment of levels of DNA methylation of known CpG regions and promoters across the genome was done with Illumina HumanMethylation 450K BeadChip and Illumina HiScan 2000. In brief, following bisulfite conversion, approximately 200 ng of the bisulfite-converted DNA per sample was used for methylation analysis. The initial quality control and identification of signal intensities for each probe were performed with Illumina GenomeStudio Software.

### Data analysis of differential methylation

The methylation data was analyzed using the Minfi package by a group-wise comparison between T21 and control samples [66]. Beta values were calculated to estimate methylation levels from the ratio of intensities between methylated and unmethylated alleles. Beta values ranged between 0 and 1, with 0 being unmethylated and 1 fully methylated. Differentially methylated probes (DMPs) were identified with dmpFinder using SWAN normalized values (betaThreshold > 0.1). The data was plotted in a Manhattan plot using the qman package in R. To avoid small methylation differences between T21 and euploid lines due to stochastic variations, we only considered differences of > 15% (average beta values of patients vs. controls) using an FDR adjusted *p* value (*q* value) and a cut off < 0.05. To investigate the chromosomal distribution of CpGs detected by DMPs, the number of hypo-and hypermethylated DMPs per chromosome was compared with the total number of 450K array probes per respective chromosome. Enrichment or depletion of CpGs with respect to chromosomal location and methylation status were calculated using data on called DMPs and Fisher’s exact test (two-sided *p* < 0.05 were considered significant).

### Bisulfite sequencing

For verification, we performed targeted analysis of DNA methylation levels of five genes annotated to CGIs with at least two DMPs (*EDNRB*, *ZNF700*, *HOXA3*, *GGCT*, and *RIBC2*) using the EpiTYPER™ technology (Agena Bioscience). Genomic DNA was treated with sodium bisulfite (EZ-96 DNA Methylation™ kit, Zymo Research, cat no.: D5004) followed by PCR using primers designated in the EpiDesigner software (epidesigner.com, Agena Biosciences; Primer sequences available upon request). Targeted regions were amplified with PCR using T7-promoter-tagger reverse primers followed by in vitro transcription. The resulting transcripts were specifically cleaved at uracil residues and subjected to MALDI-TOF analysis on an Agena Compact MassARRAY Analyzer. The EpiTYPER software 1.2.22 was used to identify the mass-fragments and for quantification of DNA-methylation at single-CpG sites or of a CpG unit. CpG sites analyzed from bisulfite-sequencing were compared with methyl-sequenced CpGs. Analysis of differential methylation was calculated using unpaired *t* test, following the Mann-Whitney *U* test (*p* < 0.05 was considered significant). Bisulfite-sequencing data was also used to generate DNA methylation profiles of five genes followed by two-way ANOVA.

### RNA extraction and quantitative RT-PCR

Total RNA was extracted from cell lysates with the RNeasy® Mini Kit (Qiagen, cat no.: 74104) following the manufacturer’s instructions. DNase I treated total RNA samples (1 μg) were subject to first-strand DNA synthesis by High Capacity cDNA Synthesis kit (ThermoFisher Scientific, cat no.: 4368814). Quantitative RT-qPCR was performed using FastStart Universal SYBR Green Master (Rox) mix (Sigma-Aldrich, cat no.: 4913850001) following manufacturer’s protocol. The reactions were performed in duplicates and run on the StepOnePlus™ Real-Time PCR System (Applied Biosystems). The expression levels of genes were measured using the primers listed below. Primer design was done with Primer3 online tool [67]. The results were normalized against *GAPDH*. The analysis was performed in StepOne Software v2.2.2 and GraphPad prism and differential expression was calculated using unpaired t-test with Welch’s correction (*p* < 0.05 was considered significant).

The expression levels of the genes were measured using the following primers:

*GAPDH* F: 5′-GTCAGCTGTTGTTGGACCTG-3′,

*GAPDH* R: 5′-GGTCACCCCATCGAAGATAC-3′,

*ZNF700* F: 5′-CACCCAGGAAGAGTGGACAT-3′,

*ZNF700* R: 5′-ATGCCTTGTGTCCAGTGTCA-3′

*HOXA*3 F: 5′-′TGCCCTTCTGATCCTTTTTG-3′,

*HOXA3* R: 5′-AATGCCAGCAACAACCCTAC-3′,

*HOXB4* F: 5′-CTGGATGCGCAAAGTTCAC-3′,

*HOXB4* R: 5′-AGCGGTTGTAGTGAAATTCCTT-3′,

### Analysis of genes associated with DMPs

For gene annotation analysis, we first mapped the CpGs detected by DMPs with respect to CGI+ regions (i.e., positioned in a CGI or in the shores or shelves of that CGI) or to open sea. We thereafter investigated if DMPs in CGI+ regions coincided with an annotated gene (UCSC; genome.ucsc.edu) according to the Illumina manifest (i.e., a CGI+ within 1.5 kb or 200 bp from a transcriptional start site (TSS1500 or TSS200, respectively), in the 5′UTR, first exon, gene body or 3′UTR, of a gene) [36]. A single gene name was annotated to each DMP using this criterion and if one or more gene name entries were annotated to one DMP the first gene was used in the enrichment analysis (Additional file 3). Probes with no UCSC RefGene name were not included in the analysis. Enrichment in functional categories was performed using the Gene Ontology (GO) knowledgebase database (geneontology.org; PANTHER Overrepresentation Test (Released 20190711)) and the Molecular Function category. Gene expression data was retrieved for DiffNPC differentiation stage from Sobol et al., 2019 [10].

## Supplementary information

**Additional file 1.** Characterization of neural cultures with trisomy 21 (DS) and euploid controls (Ctrl) at the NPC and DiffNPC differentiation stages.

**Additional file 2.** Quality control of Illumina 450K array data.

**Additional file 3.** List of all 500 DMPs called in T21 neural lines.

**Additional file 4.** Validation of DNA methylation data from 450K array analysis.

**Additional file 5.** Genes annotated to CGI+ regions that are differentially methylated in T21 neural lines.

**Additional file 6.** Validation of gene expression levels by RT-qPCR.

## Data Availability

The datasets supporting the conclusions of this article are included within the article, its additional files and, in Supplementary Table 2 of reference [10]: 12035_2019_1585_MOESM2_ESM.xls.

## Abbreviations

5mC: 5methylcytosine
CGI: CpG island
DiffNPC: Differentiated neural progenitor cell;
DMP: Differentially methylated probe
DS: Down syndrome
HSA21: Human chromosome 21
iPSC: Induced pluripotent stem cell
NPC: Neural progenitor cell
T21: Trisomy for chromosome 21
TF: Transcription factor
