# Supplementary material for: DNA methylation changes in Down syndrome derived neural iPSCs uncover co-dysregulation of ZNF and HOX3 families of transcription factors
Source: Clin Epigenetics. 2020 Jan 8;12:9. doi: 10.1186/s13148-019-0803-1 (PMC6950999; doi:10.1186/s13148-019-0803-1)

**Laan L. et al.**

**Additional File 1. Characterization of neural cultures with trisomy 21 (DS) and euploid controls (Ctrl) at the NPC and DiffNPC differentiation stages. (a)** Representative images from trisomic (DS; upper panel) and euploid (Ctrl; lower panel) neural cultures after immunofluorescent stainings of Nestin and Pax6 in NPCs (left), and of GFAP, TUBB3 and VIM in DiffNPCs (middle, right). **(b)** Heat-maps illustrating gene expression levels from RNA sequencing of trisomic (DS) and euploid (Ctrl) cultures in NPCs and DiffNPCs, respectively. Heat-maps were obtained for markers of the different neural subtypes neuroepitheleal cells, radial glia, astrocytes and neurons (GABAergic and glutamatergic). Expression of markers for oligodendrocytes and microglia were very low. Neural marker analyses from stainings and RNA sequencing suggested similar distribution of cell types in trisomic and euploid lines at both differentiation stages.


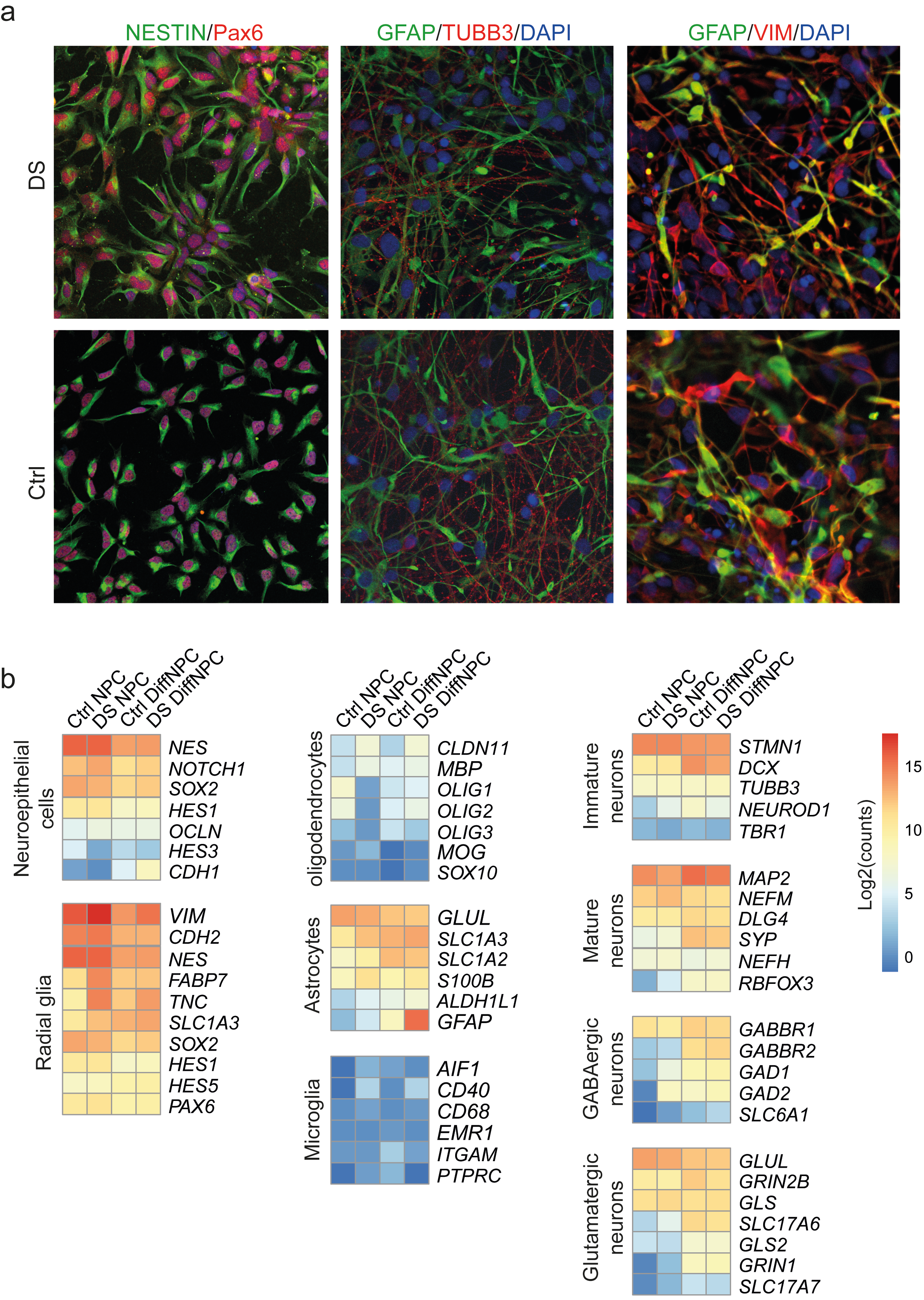

Supplement: Supplementary file 1 — Additional file 1. Characterization of neural cultures with trisomy 21 (DS) and euploid controls (Ctrl) at the NPC and DiffNPC differentiation stages. [file 13148_2019_803_MOESM1_ESM.docx]
