# Supplementary material for: DNA methylation changes in Down syndrome derived neural iPSCs uncover co-dysregulation of ZNF and HOX3 families of transcription factors
Source: Clin Epigenetics. 2020 Jan 8;12:9. doi: 10.1186/s13148-019-0803-1 (PMC6950999; doi:10.1186/s13148-019-0803-1)

**Laan L. et al.**

**Additional File 2. Quality control of Illumina 450K array data. (a)** Beta density plot of methylation beta values of sample groups coloured by sample group. **(b)** Bean blot showing the distribution of beta values for each sample in its own section, revealing a bimodal distribution of data without outliers. Ctrl: Euploid neural cultures at the NPC and DiffNPC stages of differentiation (red); DS: Trisomic neural cultures at the NPC and DiffNPC stages of differentiation (blue).


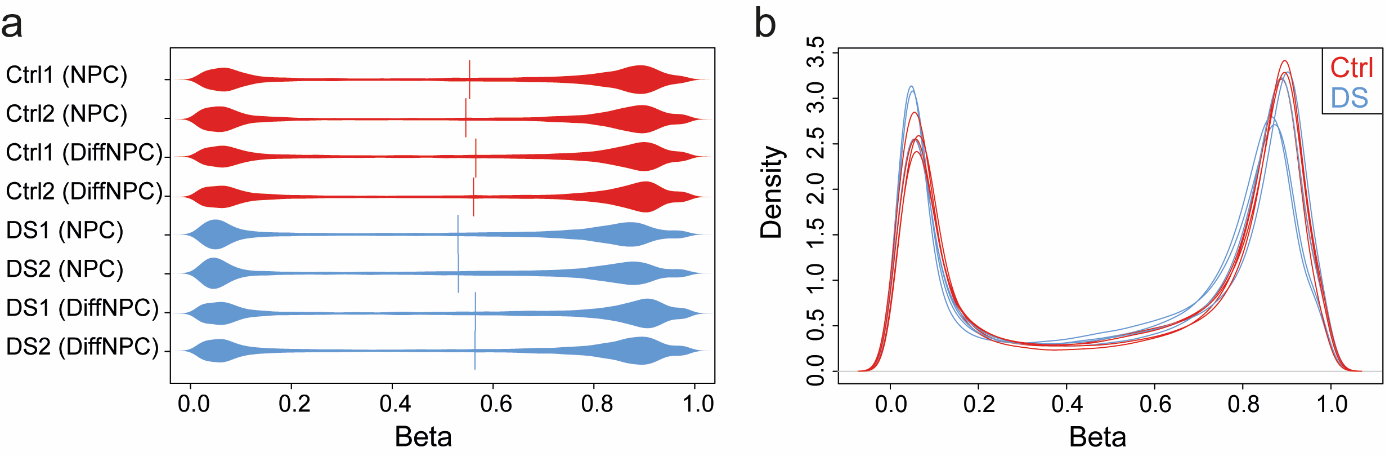

Supplement: Supplementary file 2 — Additional file 2. Quality control of Illumina 450K array data. [file 13148_2019_803_MOESM2_ESM.docx]
