# Supplementary material for: DNA methylation changes in Down syndrome derived neural iPSCs uncover co-dysregulation of ZNF and HOX3 families of transcription factors
Source: Clin Epigenetics. 2020 Jan 8;12:9. doi: 10.1186/s13148-019-0803-1 (PMC6950999; doi:10.1186/s13148-019-0803-1)

**Laan L. et al.**

**Additional File 4.** **Validation of DNA methylation data from 450K array analysis**. Methylation of individual CpGs measured by two different methods, **(a)** Illumina 450K array and **(b)** targeted EpiTYPER sequencing, in euploid (Ctrl; red) and T21 (DS; blue) neural cells for 8 CpGs. The methylation status of CpGs queried by the array probes cg07495027 and cg12120741 (*EDNRB* gene); probe cg22610787 (*ZNF700* gene); probe cg14573448 (*HOXA3* gene); probes cg23780937 and cg12881726 (*GGCT* gene); probes cg03780455 and cg20078119 (*RIBC2* gene). Each dot represents one sample. A consistent methylation pattern was confirmed for each CpG analyzed with both assays. *P* values were calculated by unpaired t-test using the Mann-Whitney U test. **(c)** Continuous DNA methylation of five selected regions obtained with the EpiTYPER assay; the N-Shore of *ERNRB*, the N-shore of *ZNF700*, S-Shore of *HOXA3*, the island of *GGCT* and the island of *RIBC2*. Two-way ANOVA was used to identify interaction differences, *p* values are indicated by stars (*, p<0.05: ***, p<0.001).


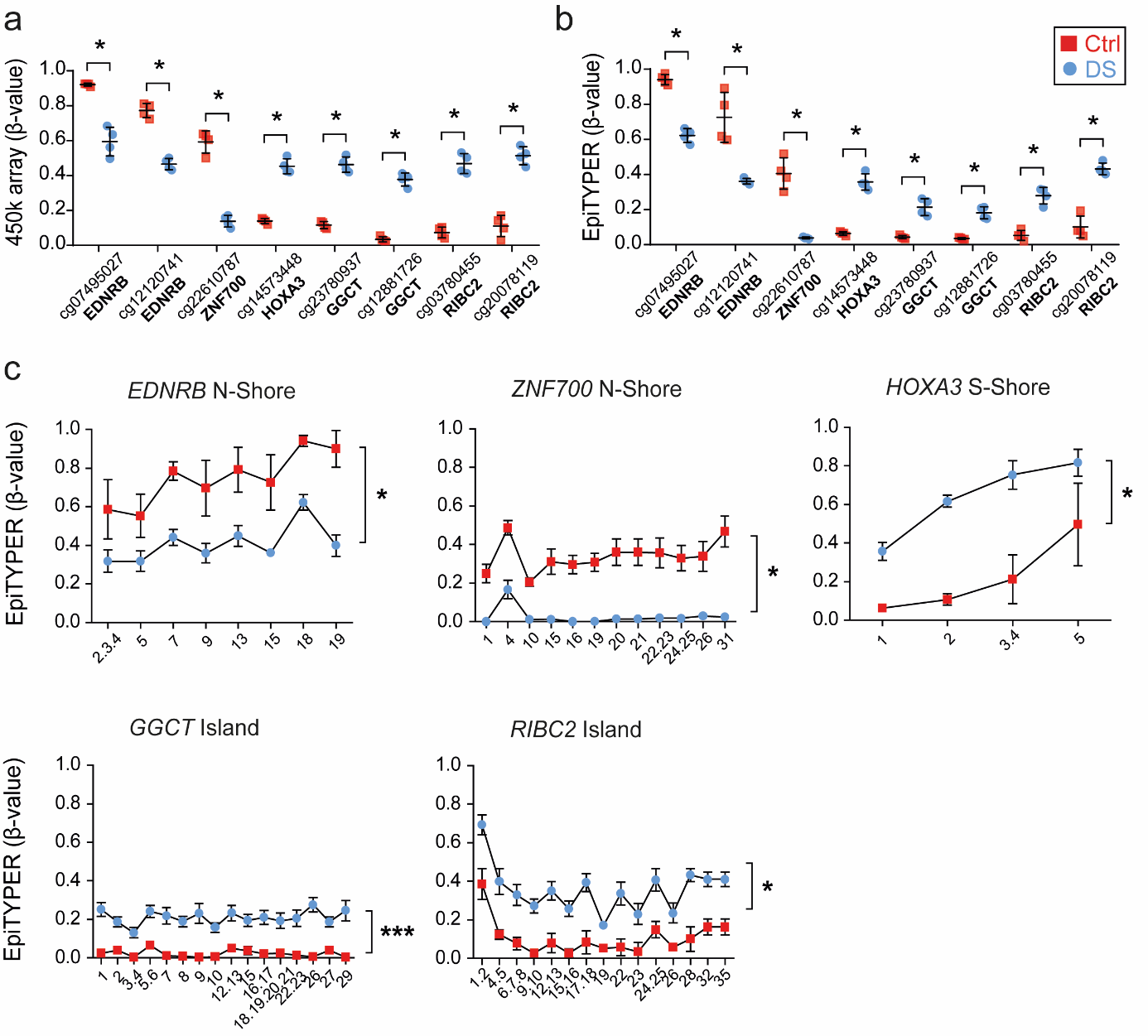

Supplement: Supplementary file 4 — Additional file 4. Validation of DNA methylation data from 450K array analysis. [file 13148_2019_803_MOESM4_ESM.docx]
