# Supplementary material for: DNA methylation changes in Down syndrome derived neural iPSCs uncover co-dysregulation of ZNF and HOX3 families of transcription factors
Source: Clin Epigenetics. 2020 Jan 8;12:9. doi: 10.1186/s13148-019-0803-1 (PMC6950999; doi:10.1186/s13148-019-0803-1)

**Laan L. et al.**

**Additional File 6. Validation of gene expression levels by RT-qPCR.** Three genes (*ZNF700*, *HOXA3* and *HOXB3*), belonging to the DNA binding category (GO) after enrichment analysis, were selected for validation of differential expression in DiffNPCs (indicated by RNA sequencing data). Bars represent the log10 fold-change values of expression levels in two biological replicates and their technical replicates (n=4). The mRNA levels were normalized to the expression of *GAPDH*. Statistical analysis was performed in Prism using unpaired t-test with Welch’s correction.


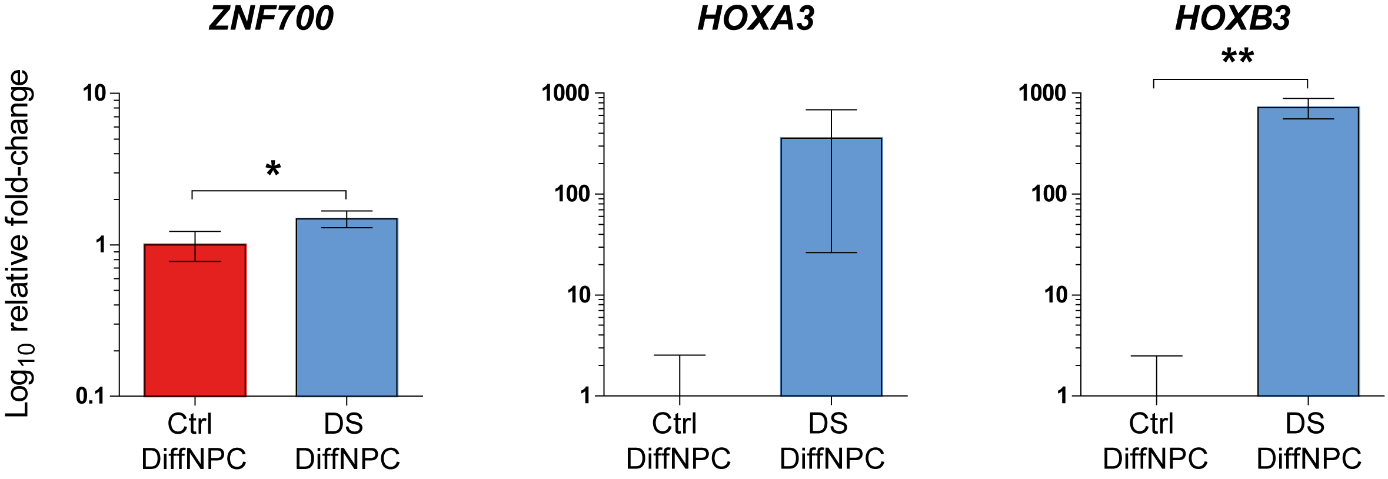

Supplement: Supplementary file 6 — Additional file 6. Validation of gene expression levels by RT-qPCR. [file 13148_2019_803_MOESM6_ESM.docx]
